# 

**Published:** 1886-10

**Authors:** 


### CONTENTS.

### COMMUNICATIONS.

Post-Graduate Study.................................................... 479
Is Dentistry a Profession?.............................................. 491
Proper Care of Poor Children's Teeth.............................. 498
Deafness Cured by Excision of Tonsils.............................. 503

### PROCEEDINGS.

National Association of Dental Faculties.......................... 505
National Association of Dental Examiners........................ 508

### SELECTIONS.

Aluminium Bronze in Medicine and Dentistry..................... 508
The Influence of Beer, Tobacco, etc., Upon Digestion.......... 511
A Tooth Growing in the Nose........................................ 512
Two Cases of Swallowing False Teeth.............................. 513
The Spirit of Imitation in Medicine................................. 513
Thinking and Working................................................. 514
Diseases Peculiar to Bakers.......................................... 514
Purity of Drinking Water............................................. 515
SALMAGUNDI ............................................................ 516

### EDITORIAL.

The International Medical Congress................................ 527
Ohio State Dental Society.............................................. 528
Bibliographical........................................................... 529

